# Automated classification of three-dimensional reconstructions of coral reefs using convolutional neural networks

**DOI:** 10.1371/journal.pone.0230671

**Published:** 2020-03-24

**Authors:** Brian M. Hopkinson, Andrew C. King, Daniel P. Owen, Matthew Johnson-Roberson, Matthew H. Long, Suchendra M. Bhandarkar

**Affiliations:** 1 Department of Marine Sciences, University of Georgia, Athens, Georgia, United States of America; 2 Institute for Artificial Intelligence, University of Georgia, Athens, Georgia, United States of America; 3 Department of Naval Architecture and Marine Engineering, University of Michigan, Ann Arbor, Michigan, United States of America; 4 Marine Chemistry and Geochemistry Department, Woods Hole Oceanographic Institution, Woods Hole, Massachusetts, United States of America; 5 Department of Computer Science, University of Georgia, Athens, Georgia, United States of America; University of Guam, GUAM

## Abstract

Coral reefs are biologically diverse and structurally complex ecosystems, which have been severally affected by human actions. Consequently, there is a need for rapid ecological assessment of coral reefs, but current approaches require time consuming manual analysis, either during a dive survey or on images collected during a survey. Reef structural complexity is essential for ecological function but is challenging to measure and often relegated to simple metrics such as rugosity. Recent advances in computer vision and machine learning offer the potential to alleviate some of these limitations. We developed an approach to automatically classify 3D reconstructions of reef sections and assessed the accuracy of this approach. 3D reconstructions of reef sections were generated using commercial Structure-from-Motion software with images extracted from video surveys. To generate a 3D classified map, locations on the 3D reconstruction were mapped back into the original images to extract multiple views of the location. Several approaches were tested to merge information from multiple views of a point into a single classification, all of which used convolutional neural networks to classify or extract features from the images, but differ in the strategy employed for merging information. Approaches to merging information entailed voting, probability averaging, and a learned neural-network layer. All approaches performed similarly achieving overall classification accuracies of ~96% and >90% accuracy on most classes. With this high classification accuracy, these approaches are suitable for many ecological applications.

## Introduction

Coral reefs are biologically diverse and structurally complex ecosystems [1, 2]. While these characteristics are a large part of what makes coral reefs a topic of scientific and cultural interest, they also make it technically challenging to study these ecosystems. Assessing the abundance and distribution of species on a reef, the most fundamental descriptor of the reef’s state, requires a substantial investment of time, either during a diver survey or annotating images later. This limits the spatial and temporal scales over which ecological surveys can be conducted and so hinders identification of factors driving reef community structure and ecosystem health. However, recent rapid advances in the quality and availability of computer vision and machine learning tools have gradually been removing some of these barriers.

In reef ecosystems, the most common metric for species abundance is percent cover: the percentage of surface occupied by a given taxa or substrate when viewed from overhead (e.g. [3, 4]). This metric is generally assessed by annotating randomly selected points in an image and determining the relative frequency of various classes. As this metric is time consuming to compute but frequently assessed, several steps toward the automation of the computational procedure and related tasks have been taken. The first steps were the development of programs that automatically select random locations, facilitate image viewing, and track annotation frequency [5], and such approaches have been widely adopted by the research community. More recently progress has been made in training automated classifiers, such as Support Vector Machines (SVMs) and Convolutional Neural Networks (CNNs), to estimate percent cover. Beijbom et. al [6, 7] trained SVMs to classify points in reef images based on local texture and color features, and obtained promising results for most classes (> 60% accuracy), which were resolved to genus or functional group level. This framework has been incorporated into an online tool for the community (coralnet.ucsd.edu) and updated to use CNNs, which improved the system’s performance [8]. Working in a more temperate habitat, Friedman [9] explored the ability of different classifiers (SVMs, k-nearest neighbor, decision trees) to segment images into different functional groups based on texture, color, and shape features. The best performing classifier (SVM) had high classification accuracy on abundant classes (>80%), but its accuracy declined substantially on less common classes. Recently, CNNs have shown promise for segmentation of coral reef images [10], and determining cover of different taxa on reefs [11–13]. A number of related approaches have also been reported, and attest to the continued progress in automated image annotation [14–16]. These initial steps in automating image classification have been promising and with the advent of CNNs, which have dramatically improved classification accuracy in other domains, automated classification of reef images may soon become routine.

CNNs are deep (many-layered) neural network-based classifiers that use convolutional filters to extract features from image data, gradually forming higher-level representations of the image in the network’s upper layers [17]. Convolutional filters have long been used to extract image features, but importantly in CNNs the filters are adapted (“learned”) to best classify the particular dataset they are trained on. These learned filters combined with the deep structure of the network are thought to be key to the dramatic improvements in classification accuracy obtained by CNNs compared to older methods in which hand-engineered features (e.g. Gabor filters, linear binary patterns) are fed into machine learning classifiers (SVMs, decision trees, etc) [18]. CNNs have been applied in a wide range of applications from face recognition to identification of actual neurons in microscopic images [19, 20]. In the ecological domain, CNNs have been applied in diverse settings such as detection of insects, wildlife in terrestrial ecosystems, and scallops on the sea floor [21–23].

Historically coral reefs have often been treated as two-dimensional systems (e.g. percent cover), but structurally complex coral reefs are most accurately studied in three dimensions (3D). Structural complexity at small to medium spatial scales (~10 cm to 10 m; typically assessed by a rugosity index) is linked to the high diversity of organisms on coral reefs and to ecosystem services, including coastal protection and fisheries [2]. Highly complex frameworks create numerous microhabitats that vary in terms of light intensity, exposure to flow, and protection from predators, among other ecologically relevant characteristics. These microhabitats provide opportunity for niche divergence, increasing the diversity of reef ecosystems [24, 25]. The spatial complexity of the intricate, interwoven reef framework is not fully captured by classic methods to measure complexity such as the chain-tape rugosity method. Here too computer vision approaches are providing new tools to capture the structural complexity of reef ecosystems. 3D reconstructions of reefs can be generated from images using Simultaneous Localization and Mapping (SLAM) or Structure-from-Motion (SfM) techniques [26, 27] and employed to assess structural complexity in a more comprehensive fashion [28–31]. 3D reconstructions have also been used to provide better insight into spatial clustering of species on reefs [32], the effects of disturbances on reef complexity and community structure [33], and to study disease prevalence and spatial distribution [34].

Despite parallel lines of research into automated classification of reef images and the use of images to generate 3D reconstructions of reefs, automated classification techniques have not yet been applied to classify 3D reef reconstructions. Classified 3D maps of coral reefs have the potential to provide new insights into the spatial relationships among taxa and offer more realistic representations of the biomass of organisms in the system compared to two-dimensional metrics such as percent cover. Such maps have been successfully produced manually and showed great promise [32], but manual construction is extremely labor intensive. Here, we report a method to produce classified 3D maps using CNNs to classify points on 3D reconstructions generated using commercial SfM software.

## Materials and methods

### Image acquisition

Images were acquired on Little Grecian (25.1185°N, 80.3005°W) and Horseshoe (25.1393°N, 80.2945°W) reefs in the Florida Keys using a stereo-video camera (Dual GoPro HERO3+ Black) enclosed in an underwater housing with a flat view port. A swimmer swam 1–3 m above the reef in a lawnmower pattern while recording 2.7k (2704 x 1524) video at 30 frames per second on “medium” field of view setting. While GoPro cameras are known for their wide field of view imparting substantial non-linear distortions, the combination of the narrower field of view setting (“medium”) and flat port water-air interface, which acts as a focusing lens element, resulted in a moderate field of view (60° in the horizontal direction) and minimal non-linear distortions. Images (video frames) were then extracted from the videos at 1–4 frames per second with higher rates used in videos where the swimmer was moving faster or the structure was changing more rapidly. Frame extraction rates were chosen to achieve high overlap between successive images (>70%), which is necessary for successful 3D reconstruction. The surveys generally covered approximately 10 m x 10 m patches of reef, though the actual area imaged varied depending on depth of the reef, water conditions, and the ability of the swimmer to maintain overlap between survey lines.

### Generation of 3D reconstructions

3D reconstructions were generated from images using commercial SfM software (Agisoft Photoscan 1.4.3, now Metashape) following methodology similar to Burns et al. [27]. Images from only one of the two stereocameras were used in the SfM reconstruction because the stereocamera baseline was too small (33 mm) for the program to handle (though note that there was sufficient stereo-disparity for custom methods to make use of, see below). This produced a 3D reconstruction with unknown scale. The scale was subsequently determined and the reconstructions converted to an absolute scale using the stereo-images via a custom procedure described below. Agisoft Photoscan was used for all steps of the 3D reconstruction process other than scale determination.

The SfM reconstruction procedure begins first by automatically identifying common points viewed in different images. These common points are then used to determine camera locations and to build a sparse point cloud representing points on the reef. Between 100 and 5000 images were used in each reconstruction (Table 1). Subsequently, the point cloud was expanded by identifying additional matching patches between images to produce a dense point cloud (“Medium” quality was used). A triangular mesh was then generated from the dense point cloud (“Medium” density), producing roughly 200k-3000k triangular faces to represent the surface of the reef (Table 1). A texture map was produced from the images for visualization.

**Table 1:** 3D reconstruction statistics.

ID	Reef Site	Year/Month	Group	Images	Mesh Elements (thousands)	Avg. Element Size (cm)	Surface Area (m^2^)	Mean Error (pixels)	calibra-tion R^2^
LG1	LG	2015/7	1	113	237	4.0	143	1.32	0.92
LG2	LG	2015/7	1	115	228	4.6	181	1.85	0.89
LG3	LG	2017/7	2	279	414	3.6	205	2.39	0.97
LG4	LG	2017/7	2	451	917	2.9	288	1.54	0.98
LG5	LG	2017/12	3	422	525	4.2	353	3.41	0.96
LG6	LG	2017/12	3	487	576	3.5	271	3.05	0.96
LG7	LG	2017/12	3	406	418	3.4	184	2.97	0.97
LG8	LG	2017/12	3	523	660	3.3	266	2.95	0.99
LG9	LG	2018/6	4	5019	3000	4.5	2349	2.08	--
H1	H	2015/7	5	417	551	2.9	180	2.76	0.96
H2	H	2015/7	5	196	1331	1.6	132	2.51	0.95

To provide an absolute scale for the reconstructions, absolute distances between points were calculated in individual stereo-image pairs and compared with relative distances between these points in the 3D mesh, similar to a previously described approach [35]. First, the 3D image points in selected stereo-pairs were determined from a disparity map [36] and the camera calibration parameters [37]. Absolute distances between ten selected points (Scale Invariant Feature Transform, SIFT, feature points in the images) were calculated in each stereo-pair. These same points were then located in the 3D mesh by mapping SIFT points in the images to the mesh using the camera transformation matrices for each image produced in the SfM procedure. Relative distances were then calculated between these 3D mesh points. A scale parameter was determined by fitting a line to estimate a relationship between the absolute and relative distances. The 3D meshes were then converted to absolute distance units (meters) using this scale parameter. In three instances, a calibration target of known length was deployed within the imaging area and the measured target size in the reconstructions was similar to the expected value (91%, 87%, and 94% of the expected size). For this work, the only purpose of the absolute scale calibration is to provide an approximate size of the 3D reconstructions for presentation. Absolute scale is not needed for reprojection of mesh faces into the original images or any part of the classification procedure.

### Identifying image locations corresponding to mesh points

**Fig 1:**
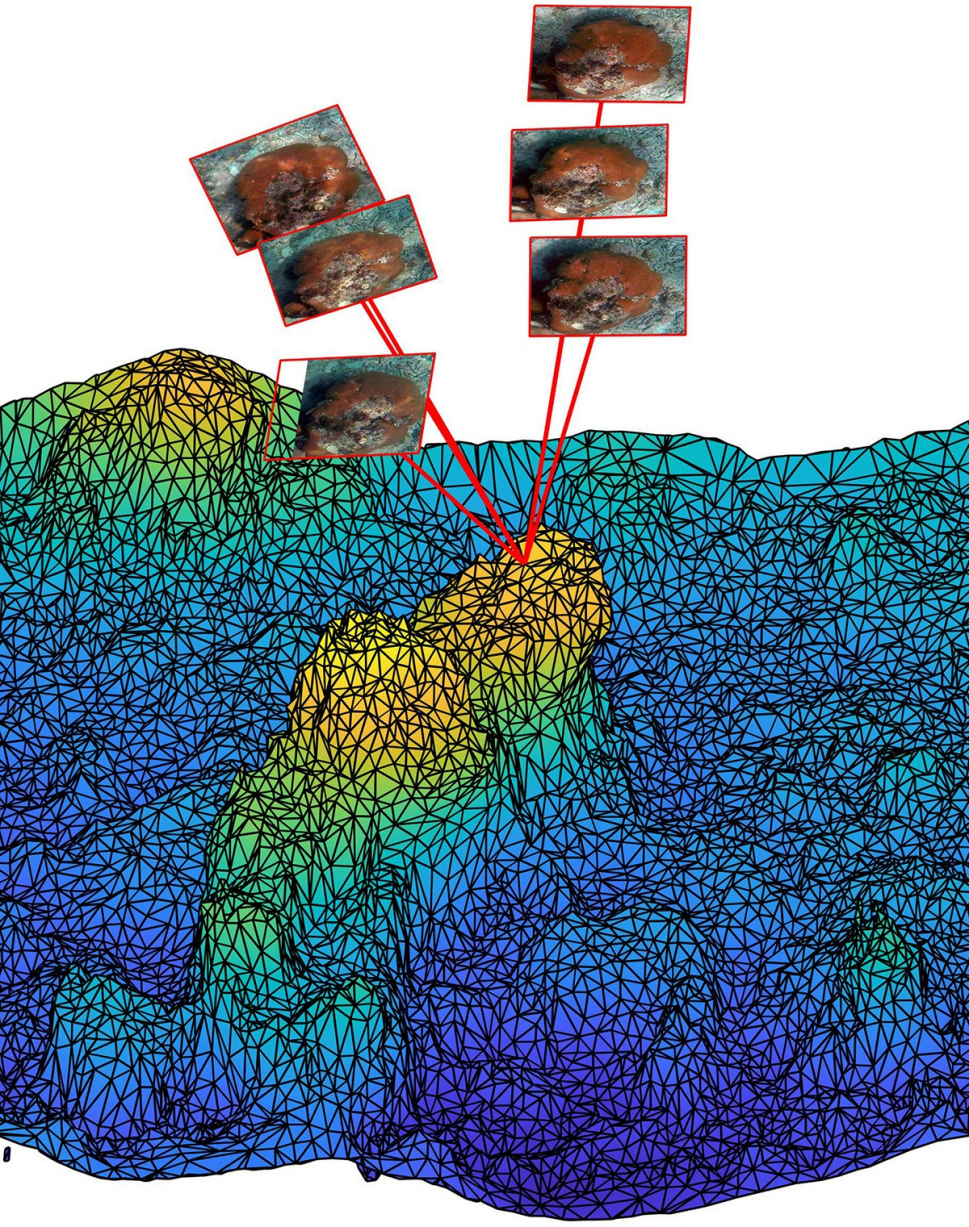
Illustration of the relationship between 3D reef reconstructions and images. 3D reconstructions are composed of linked triangular elements forming a surface mesh. The image locations where each triangular element is captured can be calculated from the camera transformation matrix and camera model as illustrated here for an element on a small *S*. *siderea* coral. The color of the mesh elements represents height above bottom.

Triangular mesh elements making up the 3D reconstructions are typically viewed in multiple images (Fig 1). To identify the image locations where each mesh element was viewed the center of each mesh element was reprojected into the images. Camera transformation matrices and camera calibration parameters were obtained from Agisoft Photoscan as part of the 3D reconstruction procedure. The camera transform matrices were inverted and used to transform mesh element centers from world coordinates to camera coordinates. Mesh element centers were then projected into cameras to determine if they were visible by applying the camera calibration model accounting for radial and tangential non-linear distortions [37]. The availability of a clear line of sight between each camera and mesh element center was checked by projecting a ray from the camera center to the element center and checking for intersections with any other mesh element. This test was accelerated using a lightweight ray-tracing library (NanoRT) that uses bounding volume hierarchies to reduce the number of ray-mesh intersection tests required. Locations where each mesh face was viewed were stored for later use in classification. On average, each mesh element was viewed in ≈ 11.9 images.

### Classes and training dataset

Data to train, validate, and test automated classifiers were produced by manual annotation of mesh element into 10 classes (Table 2). A mesh element was selected pseudo-randomly and up to 6 image patches corresponding to that mesh element were shown to an annotator. The annotator was tasked with classifying the mesh element based on the category present in the majority of the images. In most cases, all images contained the same category, but this was not always the case due to moving objects (octocorals, fish, etc) or (rarely) errors in the 3D reconstruction. In such cases, the class present in the majority of images was applied to the mesh element.

**Table 2:** Class descriptions.

Class Name	Type	Description
Algae	Functional Group	Macroalgae and/or dense turf algae
Antillogorgia	Functional Group	Antillogorgia spp. octocorals, commonly called sea plumes
*G*. *ventalina*	Species	Sea fan *Gorgonia ventalina*
Sea Rods	Functional Group	a highly varied group of octocorals including Eunicea, Plexaura, Plexaurella, Muricea, etc
*A*. *palmata*	Species	*Acropora palmata* coral
*P*. *astreoides*	Species	*Porites astreoides* coral
*S*. *siderea*	Species	*Siderastrea siderea* coral
Orbicella	Genus	*O*. *faveolata* and *O*. *annularis* corals
Rubble	Substrate	Bare coral rubble or rubble covered with crustose coralline algae or low density algal turf
Sand	Substrate	Loose sand

The 10 categories include biological classes ranging from individual species to functional groups and two substrate classes (rubble, sand). These classes encompass the vast majority of the benthic cover in the reefs under study, can be distinguished in images by human annotators, and are appropriate for our downstream application (estimation of primary production by different taxa). Alternate classes could be chosen for other purposes. The “Rubble” class represents coral rubble that is typically covered in crustose coralline algae or low density algal turf, which could not be distinguished in the images. There is some overlap between Rubble and “Algae”, which represents macroalgae and dense algal turf, but in most cases the distinction was clear. Rubble grades into Sand and though the distinction was generally clear there were ambiguous cases. A total 12,413 mesh elements were manually annotated by two different annotators in 11 data sets (S1 Table). The manually annotated mesh elements were randomly assigned to train (70%), validation (10%), and test (20%) datasets.

### Machine learning classifiers

Several approaches were tested to classify the mesh elements of the 3D reconstructions. In the computer vision literature, this process is known as “semantic segmentation”, assigning a classification to each element or pixel, but to minimize unfamiliar vocabulary this term will be avoided. In previous work on a smaller version of this dataset [38], we found that CNN classifiers out performed traditional methods (support vector machine using texture/color features) for point annotations in images, and that of the CNN architectures tested ResNets [39] performed the best. Consequently, we adopted ResNet152 as the base for all our 3D classification approaches. All deep learning work was conducted using the PyTorch framework [40] and performed on a desktop computer with an 8 core Intel CPU (i7-7820X), 64 GB of system RAM, and an NVIDIA GPU (GeForce GTX1080Ti: 3584 CUDA cores, 11 GB RAM).

First, ResNet152 was trained to classify point locations in single images from the training dataset. Image patches (200 x 200) were extracted centered on the points where each mesh element center was viewed in images. These image patches and corresponding class labels were used to train ResNet152 closely following King et al. [38]. The uppermost prediction layer of ResNet152 was replaced with a new prediction layer (fully-connected) with 10 outputs, one for each class. Initially, the parameters of the prediction layer were trained from scratch for 75 epochs using the Adam optimizer [41] with a cross entropy loss function and a learning rate of 1 x10^-4^. During this stage the lower layers weights were fixed at ImageNet pretrained values [39]. Subsequently all model parameters were trained for 50 epochs at a learning rate of 1 x10^-5^. After each training epoch, the model was checked against the validation dataset and the model with the best overall accuracy on the validation dataset was retained.

The trained ResNet152 model was used as the basis for three methods to classify mesh elements that merge information from multiple images: voting, averaging, and a neural network. For the voting and averaging methods, all image patches corresponding to a mesh element were individually passed through ResNet152. As in the initial training phase, fixed size patches (200 x 200 pixels) were extracted from images centered on the location where a mesh element center was projected into the image. In the voting scheme, each view “voted” for the class with the highest prediction probability from the ResNet152 classifier, and the class with the most votes among all views was selected as the mesh element label. In the averaging scheme, class probabilities predicted by each view were obtained by applying a softmax function to the logit prediction vector output by ResNet152. The class probabilities for all views were averaged and the class with the highest prediction probability was selected as the mesh element label. This approach provides greater weight to views in which the classifier was more confident about its prediction.

**Fig 2:**
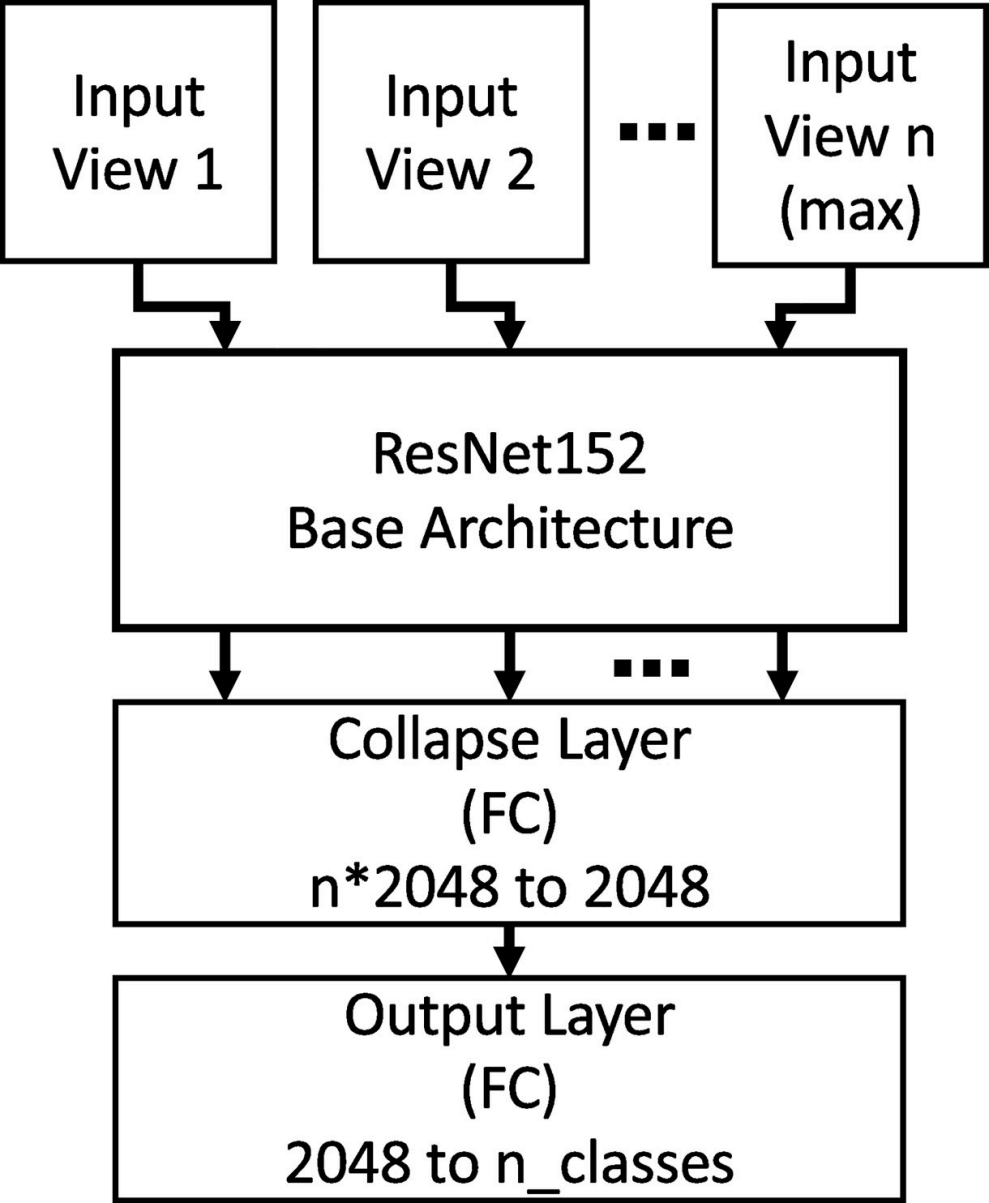
Diagram of the nViewNet neural network used to merge information from multiple views. A set number of image patches (n) of a mesh element are passed through an abbreviated ResNet152 CNN. The feature vectors summarizing each view are concatenated and passed into a fully connected (FC) collapse layer followed by an output layer, which provides a single prediction for each mesh element.

Finally, a neural network architecture (nViewNet) was developed to provide greater flexibility in how information is merged from multiple views. In this approach, each image patch is summarized as a feature vector obtained from the output of an abbreviated ResNet152 network. Feature vectors from each view are then merged in a fully-connected “collapse” layer followed by a second fully-connected layer that outputs a prediction vector (logit vector), the entries of which are related to the probability that each class is the correct prediction (Fig 2). The number of views (n) accepted by nViewNet is set during the training phase (up to n = 16 has been tested). If a mesh element has been viewed more than n times, n views are randomly selected and fed into the network. If a mesh element has been viewed exactly n times, all n views are all fed into the network. If a mesh element has been viewed less than n times, the views are repeated so that a total of n views are fed into the network. Eight views (nViewNet-8) were used in all cases unless otherwise noted since this provided high classification accuracy at reasonable speed (see below). The collapse and fully-connected layers of nViewNet were trained using the Adam optimizer at a learning rate of 1 x10^-4^ for 50 epochs, which was sufficient for convergence. The weights of the ResNet152 feature extractor were frozen at the pretrained values used in the voting and averaging methods.

All models were trained with the training dataset and the validation dataset was used to select the best performing model in the training phase, avoiding overfitting. The performance of the models was then assessed on the test dataset, which was not used at all in the training/model selection phase.

### Ethics statement

This work was conducted under Florida Keys National Marine Sanctuary Permits FKNMS-2016-042 and FKNMS-2017-035. No protected species were sampled.

## Results

### 3D reconstructions

3D reconstructions were generated from images using established procedures [27]. Generally, good results were obtained with few gaps in the surface meshes, low reprojection errors (Table 1), and visually good agreement with expected morphology. The ability to generate geometrically accurate reconstructions from underwater images has been assessed more thoroughly in previous work [30, 42]. The average length of the triangular elements making up the meshes ranged between 1.6–4.6 cm (Table 1), such that the reconstructions resolve medium scale features including coral branches and sea fan blades, but the meshes do not resolve very fine scale features such as individual coral polyps or algal filaments (though note that these are typically visible in the images and so such information is incorporated by the machine learning classifiers). Our approach to calibrating the meshes using distances obtained from stereoimage pairs performed reasonably well as gauged by the sizes of calibration targets in several meshes (91%, 87%, and 94% of the expected size), and by the R^2^ values (Table 1) for linear relationships fit to the stereopair distances vs. uncalibrated mesh distances, the slope of which was used as the calibration factor. The one exception to this was LG9 where the approach failed, possibly due to the high density of octocorals that are often inadequately reconstructed. Instead the mesh was calibrated using known dimensions of sensing equipment (eddy covariance and gradient instruments [43]) visible in the 3D reconstructions.

### Classification of 3D reconstruction elements

**Fig 3:**
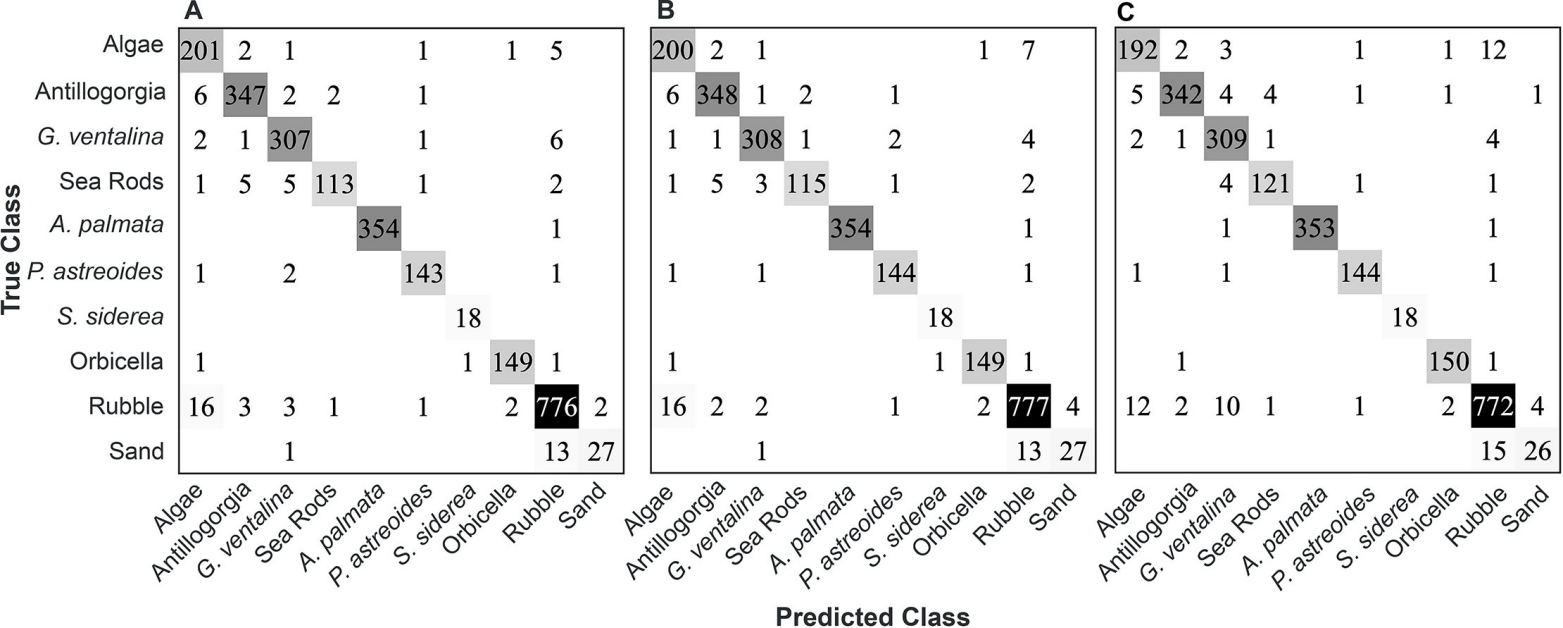
Confusion matrices showing the performance of the multi-view classifiers: (A) voting, (B) averaging, and (C) nViewNet-8. Rows represent the true class of the annotated mesh elements and the columns indicate the class predicted by the classifier. Each entry in the matrix is the number of the true class elements (row) classified into each predicted class (column).

The performance of three different approaches (voting, averaging, neural network) to classifying mesh elements in 3D reconstructions of coral reefs was assessed. The dataset used to assess performance was constructed by manually labeling 12,413 mesh elements in 11 different 3D reconstructions from two reefs in the Florida Keys. The distribution of samples was highly uneven among the classes, reflecting the distribution of these classes on the reefs, with Rubble, *Acropora palmata*, Antillogorgia, *Gorgonia ventalina*, and Algae most abundant, Orbicella, *Porites astreoides*, and Sea Rods having intermediate abundance, and Sand and *Siderastrea siderea* being relatively rare (Fig 3). The performance of the classifiers was assessed using the ‘overall classification accuracy’, the percent of all mesh elements correctly classified, the accuracy for individual classes, and the ‘balanced accuracy’. Note that we use the term ‘accuracy’ informally here for a broader audience. In the machine learning literature what is here labeled ‘accuracy’ would be referred as the ‘recall rate’ or the ‘true positive rate’ and the equivalent term in the remote sensing literature is the ‘producer’s accuracy’. Specifically, the ‘overall classification accuracy’ was calculated by summing the true positives for all classes and dividing by the total number of annotated examples in the test set. The individual class accuracies were determined as the number of true positives for a class divided by the total class count. Balanced accuracy was calculated as the average of the individual class accuracies.

The three approaches performed similarly in overall classification accuracy and on most individual classes (Fig 3). The overall classification accuracy was 96.2% using the voting scheme, 96.4% using the averaging approach, and 95.9% using nViewNet-8. The balanced accuracy was 93.6% for the voting scheme, 93.9% for the averaging approach, and 93.4% for nViewNet. All classes were classified at >90% accuracy with the exception of Sand (~65% accuracy). The sand class was rare and graded into Rubble leading to ambiguity between the classes.

**Fig 4:**
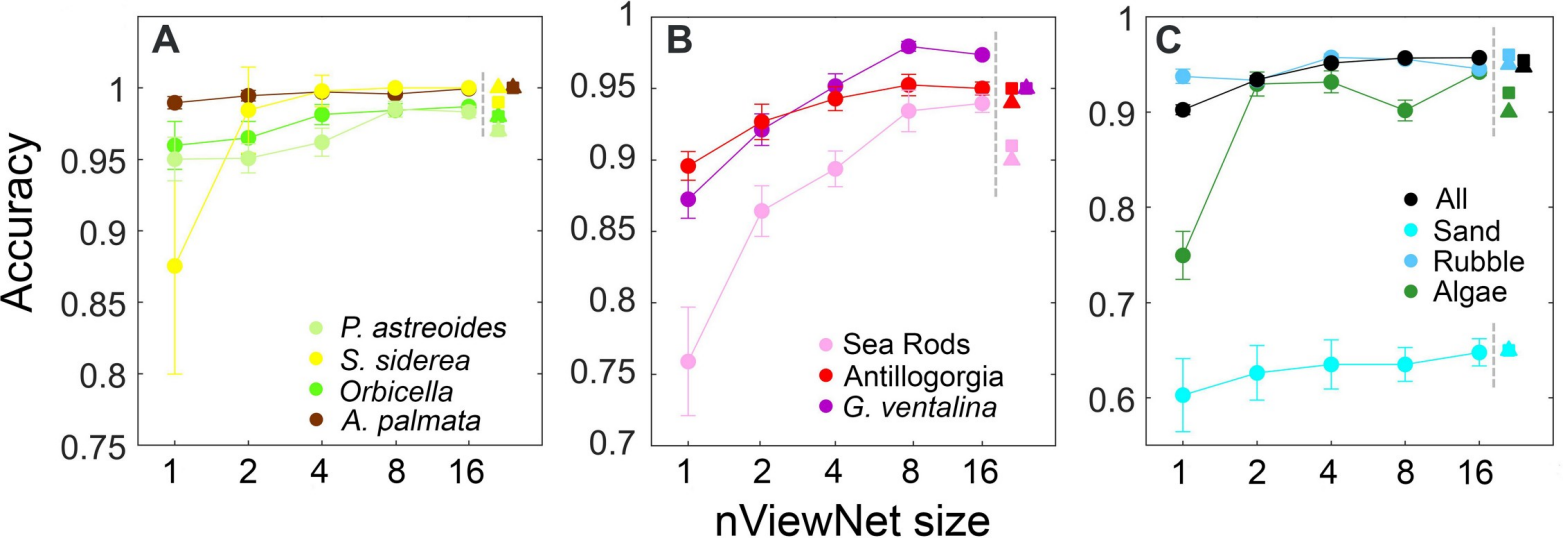
Effect of number of views input to nViewNet on classification accuracy. A) hard corals, B) octocorals, C) other classes and overall accuracy. The classification accuracies of the voting (triangles) and averaging (squares) approaches are shown to the right of the dashed vertical lines.

The usefulness of incorporating multi-view information was explored by varying the number of views used for classification in nViewNet. Although it performed similarly to the other approaches in the current classification task, nViewNet was examined further because it is expected to be a more flexible and general approach for future work. nViewNet was trained to classify mesh elements using from 1 to 16 views. The resulting nViewNet classifiers were assessed on the test set repeating the test set predictions 25 times since views are randomly selected for input into the classifier when the number of views available for a mesh element exceeds nViewNet’s capacity. Increasing the number of views from 1 to 16 led to substantial increases in prediction accuracy for many classes (Fig 4). For some classes (*S*. *siderea*, Algae) the accuracy gain came largely when increasing from 1 to 2 views, but for others (the soft coral classes: *G*. *ventalina*, Antillogorgia, Sea Rods) accuracy gain increased gradually reaching a maximum value near 8 views.

To assess the robustness and transferability of nViewNet, the model was retrained without selected datasets and then used to make predictions on these unseen datasets. For this analysis, the 3D reconstruction datasets were grouped into 5 groups based on reef location and sampling time (Table 1). The datasets within each group are very similar in terms of taxonomic composition, imaging conditions, depth, and other characteristics that would be expected to affect the performance of the machine learning classifier. Conversely, there are substantial differences in these characteristics between groups. Most obviously Little Grecian is a highly degraded reef dominated by octocorals and algae, whereas Horseshoe is dominated by *A*. *palmata*, but even within Little Grecian there are substantial differences over time and between sites imaged. Some groups are made up of sites from the back reef (Group 1, Group 3) where large boulder corals (Orbicella, *S*. *siderea*) are found, whereas others are from the reef crest/fore reef (Group 2, Group 4) where *G*. *ventalina* and *P*. *astreoides* are more common. Imaging conditions vary between sampling dates primarily due to differences in wave intensity.

For each group, the underlying ResNet152 and nViewNet-8 models were retrained excluding all data from the target group and the trained models were then used to classify mesh elements in the target group. Unsurprisingly, classification performance declined when groups were left out of model training (Table 3), but in most cases degradation was not severe. Overall classification accuracy remained at ~90% for most groups except Group 3. Group 3 was the only dataset collected in the winter when water clarity was lower and this dataset was collected a few months after Hurricane Irma, which in addition to affecting existing biota caused a bloom of red algae (Galaxaura spp.). Several classes maintained high classification accuracy including Antillogorgia, *G*. *ventalina*, and Rubble, which were all visually very similar across the datasets. Other classes showed more fragility to a lack of training data including Algae and Sea Rods. These are diverse groups that encompass taxonomically (and visually) distinct taxa depending on the dataset. For example, Halimeda were much more common on the reef crest whereas as Dictyota and Stypopodium dominated the back reef. After Hurricane Irma in the fall of 2017, Galaxaura spp. proliferated contributing to the mix of algae found on the reef.

**Table 3:** Overall and per class accuracies of leave one out tests conducted to assess model transferability. If there were <20 annotations for any class, its accuracy was not reported.

Class Name	All	Group 1	Group 2	Group 3	Group 4	Group 5
Overall	0.96	0.86	0.89	0.67	0.89	0.91
Algae	0.91	0.74	0.82	0.55	0.43	--
Antillogorgia	0.96	0.98	0.97	0.88	0.87	0.97
*G*. *ventalina*	0.97	0.86	0.84	0.88	0.95	0.97
Sea Rods	0.95	0.70	0.80	0.88	0.63	--
*A*. *palmata*	0.99	--	--	--	0.83	0.91
*P*. *astreoides*	0.98	--	0.85	0.81	0.97	1.00
*S*. *siderea*	1.00	0.78	--	0.38	--	--
Orbicella	0.99	0.96	--	0.18	0.92	--
Rubble	0.96	0.89	0.94	0.92	0.97	0.91
Sand	0.63	0.21	0.79	0.82	0.14	--

### Application to 3D reconstructions

**Fig 5:**
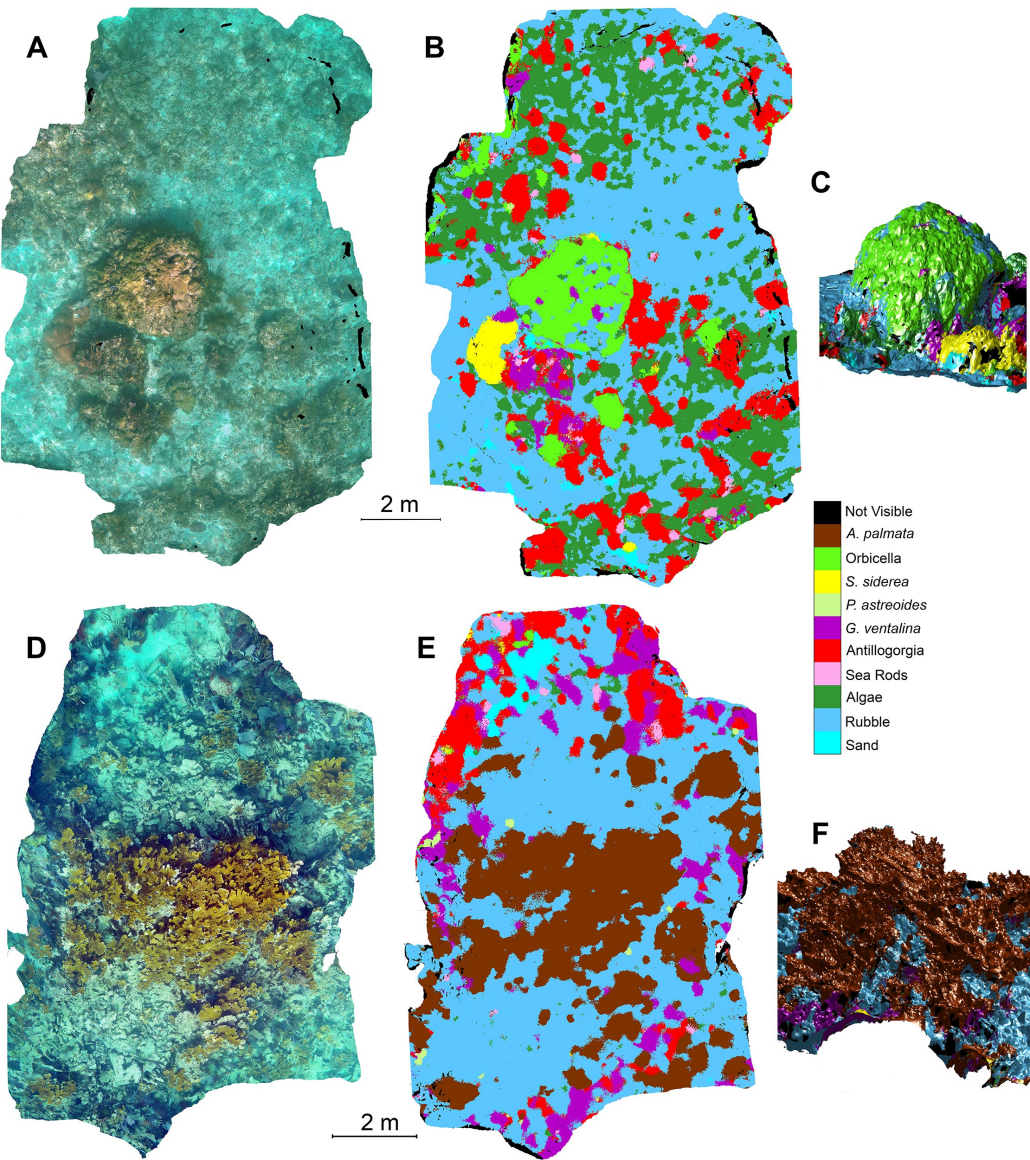
Sample texture-mapped and classified 3D reconstructions. A) Texture-mapped and B) classified reconstructions of a segment of Little Grecian reef (LG1) viewed from overhead. C) Shows a side view of the classified reconstruction with shading to highlight its three-dimensional nature. D) Texture-mapped and E) classified reconstructions of a portion of Horseshoe reef (H1). F) Shows a side-view of the classified reconstruction. 3D reconstructions were generated and texture-mapped from the original images using commercial software (Agisoft Photoscan). The 3D reconstructions were then classified using the nViewNet-8 neural network. This figure is best viewed on a computer screen.

**Fig 6:**
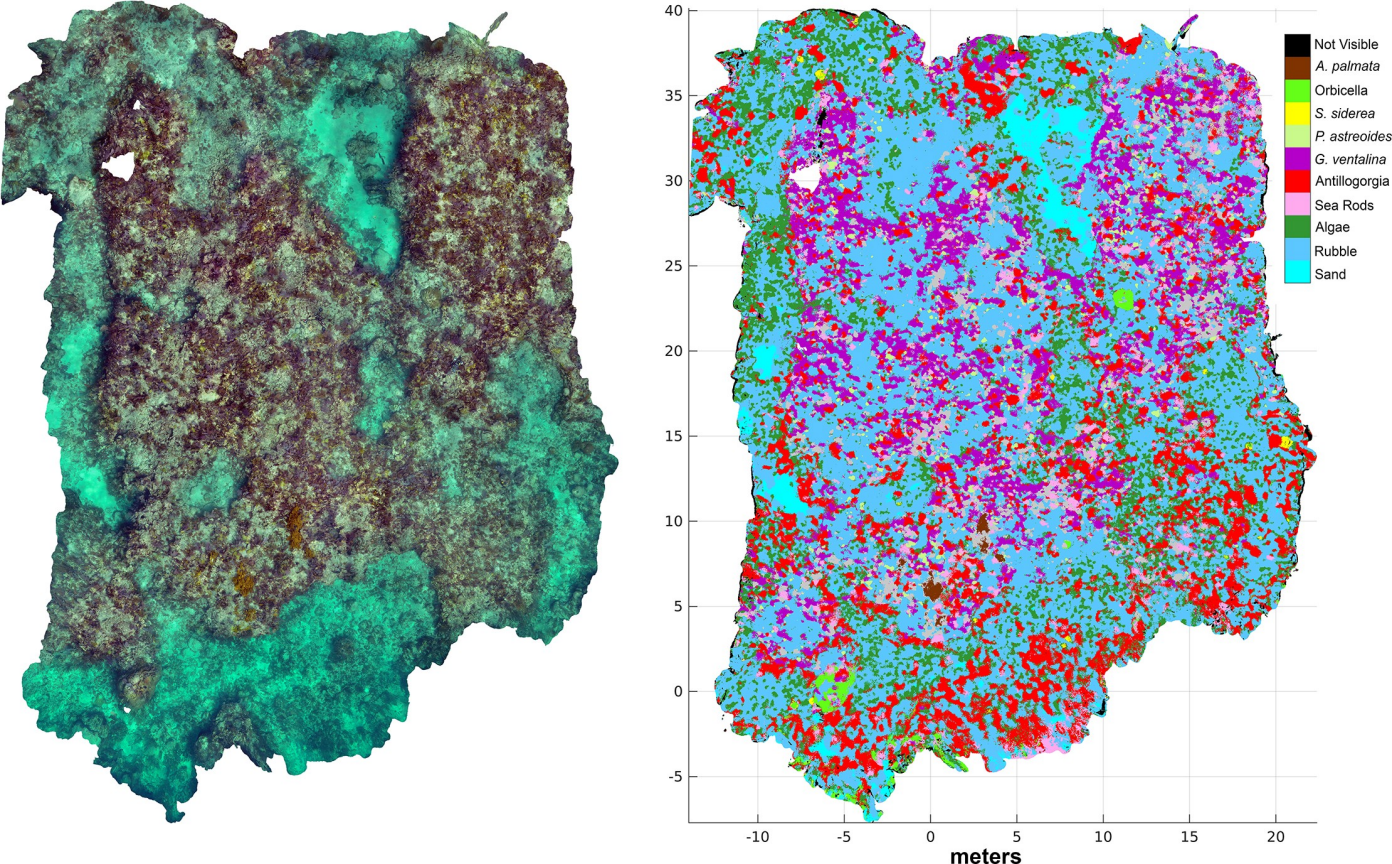
Reconstruction and classification (nViewNet-8) of a larger section of Little Grecian reef (LG9; ~40 m x 30 m) demonstrating the potential to expand the method to landscape scale. This figure is best viewed on a computer screen.

The nViewNet-8 classifier was applied to automatically label several 3D reconstructions from two reefs in the Florida Keys: Little Grecian reef, which is dominated by algae and octocorals as is typical in the Florida Keys, and Horseshoe reef, which has one of the last remaining large stands of *A*. *palmata* in the region (Figs 5, 6, S1–S6 Figs). nViewNet-8 was chosen because its performance exceeded versions using fewer views and performed comparably to nViewNet-16 but was faster and required less memory. Qualitative assessment of the classified 3D reconstructions shows good general agreement between the texture-mapped reconstructions and the classified map automatically generated using nViewNet-8. For example, in the reconstruction from Little Grecian reef in Fig 5A the large *O*. *faveolata* colony in the middle of the reconstruction and the adjacent *S*. *siderea* colony show up clearly in the automatically classified map (see S5 Fig for a close up view of this region), and the broad areas covered by rubble and algae are captured as are clumps of Antillogorgia. Similarly, in the reconstruction from Horseshoe reef, the stand of *A*. *palmata* and scattered colonies are accurately represented in the classified map as are the clumps of *G*. *ventalina* and Antillogorgia. The 3D nature of the classified maps are highlighted in Fig 5C and 5F and show that even through the photographic surveys were conducted overhead the classification works well on many of the angled surfaces. However, completely vertical surfaces and the underside of surfaces are not imaged and so cannot be classified. Some errors are evident, but most are relatively minor. For example, the regions labeled as ‘*S*. *siderea*’ in the Horseshoe reef section are instead what appears to be a red or brown algae with a very similar color to *S*. *siderea* (Fig 5E). Regions where different classes are tightly interspersed also result in problematic assignments. In this dataset, such issues typically occurred where mixed groups of octocorals and algae congregated on larger pieces of rubble or dead coral colonies (S5 Fig).

## Discussion

Coral reefs are biologically diverse and structurally complex ecosystems making them interesting but challenging systems to study. Emerging techniques in machine learning and computer vision offer the potential to provide new insights into these ecosystems. Assessing biological diversity is routine but time consuming, and machine learning techniques have recently been developed that can classify taxa in coral reef images, typically to genus-level or higher taxonomic resolution, with reasonable accuracy and significantly reduced time commitment [7, 12]. Computer vision techniques to make 3D reconstructions have also been applied to coral reef ecosystems to study their geological and biological characteristics [27, 29]. Here, we unite these two lines of research demonstrating an approach to automatically classify 3D reconstructions of sections of coral reef. The approach employs commercial SfM software (Agisoft Photoscan) to generate a 3D reconstruction from coral reef images. Views of each element in the 3D reconstruction are extracted from the images and subsequently processed by a CNN to automatically classify each element. The final product is a classified (or “semantically segmented”) 3D model of the coral reef (Fig 5). Our approach is very similar to one previously reported for labeling buildings in urban environments [44]. In coral reef environments, Pavoni et al. [45] semantically segmented orthomosaics derived from 3D reconstructions using CNNs, but the 3D information is discarded in this strategy.

3D reconstructions were generated from overlapping images of reef sections using SfM software, largely following procedures previously described and validated [27, 42]. The 3D reconstructions are surface meshes composed of linked triangular elements (Fig 1) and our ultimate goal was to classify each mesh element into one of a predetermined set of classes. In building the 3D reconstruction, the SfM algorithm determines the 3D location of the camera when each image was taken and estimates camera calibration parameters. Using this information, the image locations corresponding to each mesh element were calculated using a projective camera model that accounts for radial and tangential distortions ([37]; Fig 1). Because mesh elements were typically viewed in multiple images (on average 11.9 views per element in our data set) an approach was needed to merge information from multiple views to provide a single classification for each mesh element.

In previous work [38, 46] we assessed the ability of traditional approaches (color/texture features classified with an SVM) and different CNN architectures to classify point locations in coral reef images. This work showed that a CNN architecture (ResNet) performed best and we consequently adopted ResNets as the basis of approaches to merge information from multiple views to provide a single label for a 3D mesh element. Three information pooling methods were tested: voting, probability averaging, and a neural network, nViewNet (Fig 3). All approaches achieved high and effectively identical overall classification accuracy (~96%) and balanced accuracy (~93%). Since the neural network can learn the best way to combine information rather than using a predetermined scheme, it might generally be expected to perform better in future, more demanding classification tasks. However, the neural network is more difficult to train and use, so it is notable that the alternative schemes performed nearly as well as nViewNet in this instance. The utility of incorporating information from multiple views was clearly demonstrated by the increased prediction accuracy for many classes as the number of views input to nViewNet increased (Fig 4). An assessment of the transferability of the model (Table 3) showed nViewNet is reasonably transferable to new datasets, though it clearly benefits from having some training data from the target dataset. The extent of transferability is of course dependent on how visually similar the new dataset is to the training data. For example, datasets obtained in lower-visibility conditions and after a hurricane impacted the reef (Table 3, Group 3) were difficult to classify without training data obtained in those conditions.

The high overall classification accuracy suggests this method will be suitable for many ecological applications including community structure analysis and ecological monitoring. The classes used here range from individual species, to functional groups, and up to broad substrate categories. The classes include many of the functional groups and several hard coral species tracked by ecological monitoring programs in the Florida Keys (e.g. [3]). These categories were developed for a specific downstream application (assessment of primary production on reefs) with prior knowledge that it would be necessary to distinguish these classes in images. Consequently, the particular classification task assessed here is likely somewhat less demanding than others that may arise, for example if an ecological monitoring program was trying to replace human annotators with a machine learning algorithm without modifying the categories under study. Ultimately, the required performance of a machine learning system will depend on the specific scientific goals of a project. For example, our method should be immediately applicable to assessing the distribution and abundance of *A*. *palmata*, a threatened coral in the Caribbean, since its distinct color and visual texture allow it to be easily separated from other categories. One the other hand, a study focusing on sea rods (Plexaurella, Eunicea, etc) would need to further refine the methodology before proceeding as we were unable to routinely distinguish among genera in the images.

One inherent difficultly in studying non-rigid taxa such as octocorals is that they move over time and so are imaged at different 3D locations, violating the SfM algorithm’s assumption that the scene is static. In such cases, the images of a mesh element may be an octocoral in some images but may later be the underlying substrate as the octocoral moves with waves or currents. Many of the octocorals in our data set tended to occur in clumps, especially Antillogorgia and *G*. *ventalina*, alleviating this issue somewhat, but for the sea rod taxa, which did not generally cluster at high densities, motion contributed to the difficulty in accurately classifying this group.

This approach to classify 3D constructions shows great promise, but modifications at all phases of the pipeline could be made to improve the final classified 3D reconstructions. In the image acquisition phase, this study used a single swimmer typically navigating without aid, which limited the spatial extent of the area covered. Generating high-quality 3D reconstructions requires continuous overlap of images both along the swim track and between adjacent track lines, which is challenging for an unaided swimmer. One option is to use multiple divers to help the photographer stay oriented [47], the drawback being the additional manpower required. A more recent method uses a line attached to a fixed drum allowing a single swimmer to accurately travel in a spiral pattern, though the area covered is not dramatically larger than what an unaided swimmer is typically capable of covering [48]. We found it useful to deploy underwater marker buoys as reference points when attempting larger area surveys. Robotic platforms are capable of accurately traversing large areas and have been used to visually map extensive sections of the benthos [49, 50]. Currently such robots are quite expensive, but cheaper yet capable remotely operated underwater vehicles (ROVs) and autonomous underwater vehicles (AUVs) are becoming more widely available. These robots could also be programmed to obtain images of the environment from multiple angles to increase coverage of vertically oriented surfaces and minimize mesh deformation that arises from predominantly vertical imagery [51]. The approach described here is capable of generating landscape scale maps as shown in Fig 6, if overlapping images are acquired of the entire region.

The 3D reconstructions captured the major features of the reef structure and obtained an average mesh dimension of 1.6–4.6 cm. This resolution should be sufficient for many ecological tasks since most of the major benthic organisms (corals, octocorals, algae) are substantially larger. However, it does not resolve fine scale feature such as individual lobes of sea fans or branches of octocorals preventing detailed examination of their morphology. Finer mesh reconstructions could be generated with the existing programs but require more computational power to process. Highly resolved meshes would require higher resolution images from better cameras (e.g. still-image, global shutter cameras) and would likely need to be acquired very close (<1 m) to the benthos. However, the accuracy of 3D reconstructions from underwater images is ultimately limited by optical constraints due to the water-air imaging interface [52].

One of the most time-consuming steps in developing an accurate machine learning classifier is obtaining large amounts of training data. We hand annotated all the training data used in this study, but acceleration of this process is possible by incorporating unsupervised learning, for example by using occasional hand annotation to guide an unsupervised data clustering procedure [53]. The 3D reconstructions were built using an SfM algorithm that does not incorporate additional information about relationships between the images (order, orientation, etc) into the reconstruction process limiting the spatial scale and in some cases the quality of the reconstruction. Simultaneous Localization and Mapping (SLAM) frameworks can make use of such information (GPS position, IMU-based orientation, velocity, etc) reducing the computational complexity of the reconstruction problem and increasing the potential spatial coverage and accuracy [49, 54]. Frameworks that incorporate the ability to model moving constituents, such as non-rigid SfM or articulated parts models [55, 56], would enable more realistic representation of flexible benthic organisms such as octocorals.

Finally, the CNN architecture used here (ResNet152) worked very well, but neural network architectures continue to mature and new developments may improve classification performance [57, 58]. Additionally, there is scope for using 3D structural features to aid classification. Previous work on coral reef image classification showed that stereo-disparity (inversely proportional to depth) information provided a boost in performance for CNN-based semantic segmentation [46]. Incorporating smaller-scale structural texture derived from stereo-image pairs would into out current pipeline would be fairly straightforward. It would be particularly interesting, but more challenging, to draw on larger-scale or global structural information, capturing notions such as coral colony shape or size.

While there is room for improvement, advances in 3D reconstruction techniques and machine learning now permit mapping and automated classification of coral reefs with reasonable accuracy facilitating rapid ecological assessment and monitoring. This new approach provides reliable spatially-localized information on reef structure and species composition in contrast to current approaches (e.g. percent cover) that average information over an entire site, facilitating spatial ecology at smaller scales than previously possible. Moving from 2D metrics commonly employed now to 3D metrics enabled by methods such as ours offers more realistic representations of coral reefs. For example, a vertically-oriented macroalga or octocoral may contribute little to percent cover but have a large biomass relevant to reef metabolism, food webs, and other ecological processes.

## Conclusions

The 3D reconstruction and automated classification method described here performed well at our study sites in the Florida Keys. To obtain high quality, large-scale 3D reconstructions we found it was necessary to have continuous, high-overlap (>70%) between images and good visibility as others have noted [27]. The performance of the classifier will likely be highly dependent on the taxonomic resolution desired and the quality and scale of the images. In this study, our images were typically 1–3 meters above the reef in order to cover a larger spatial area, sacrificing small scale details necessary for finer scale taxonomic resolution. The classifier was highly accurate at distinguishing the broad classes and visually distinct species delineated in this study, which will ultimately be used in a study assessing the contribution of different taxa to productivity on these reefs. However, machine learning classifiers inevitably have more difficulty resolving finer taxonomic distinctions (e.g. [6]). To facilitate the use of this method by other researchers we have made the classifier and reprojection code publicly available (https://github.com/bmhopkinson/reef_multi-view_classification), and the associated data is publicly available (https://datadryad.org/review?doi=doi:10.5061/dryad.r465755).

## Supporting information

S1 Table Manual annotations by site. (DOCX)

S1 Fig Texture mapped (left) and classified (right) overhead views of site LG1. The reconstruction was classified using nViewNet-8.
(JPEG)

S2 Fig Texture mapped (left) and classified (right) overhead views of site LG3.
(JPEG)

S3 Fig **Texture mapped (upper) and classified (lower) overhead views of site LG4.** The reconstruction was classified using nViewNet-8.
(JPEG)

S4 Fig **Texture mapped (left) and classified (right) overhead views of site H2.** The reconstruction was classified using nViewNet-8.
(JPEG)

S5 Fig Selected region of LG1 focused on the large *O*. *faveolata* colony and adjacent *S*. *siderea* colony. The automatic classification procedure accurately delineates the Orbicella and S. siderea colony, including distinguished live tissue from dead skeleton (labeled ‘Rubble’). The clusters of mixed octocorals and algae in the lower portion of the section (e.g. just to the right of the *S*. *siderea* colony) are more difficult to classify and result in fragmented predictions in some cases.
(JPEG)

S6 Fig Selected region of LG4 showing the classifier is able to handle relatively complex, patchy regions as long as the patches are substantially greater in size than individual mesh elements. (JPEG)
