# Supplementary material for: Automated classification of three-dimensional reconstructions of coral reefs using convolutional neural networks
Source: PLoS One. 2020 Mar 24;15(3):e0230671. doi: 10.1371/journal.pone.0230671 (PMC7093084; doi:10.1371/journal.pone.0230671)
Supplement: S1 Table — (DOCX) [file pone.0230671.s001.docx]

Supplemental Table 1. Manual Annotations by Site

| **ID** | **Algae** | **Antil** | **Gvent** | **SRod** | **Apalm** | **Past** | **Ssid** | **Orb** | **Rub** | **Sand** | **All** |
| --- | --- | --- | --- | --- | --- | --- | --- | --- | --- | --- | --- |
| LG1 | 119 | 77 | 8 | 21 | 0 | 3 | 33 | 85 | 435 | 51 | 832 |
| LG2 | 95 | 100 | 41 | 6 | 0 | 1 | 21 | 145 | 264 | 1 | 674 |
| LG3 | 262 | 356 | 172 | 22 | 0 | 164 | 6 | 0 | 254 | 45 | 1281 |
| LG4 | 257 | 237 | 137 | 23 | 0 | 149 | 8 | 1 | 260 | 17 | 1089 |
| LG5 | 30 | 79 | 31 | 24 | 2 | 0 | 1 | 144 | 140 | 18 | 469 |
| LG6 | 67 | 78 | 63 | 50 | 0 | 15 | 4 | 178 | 166 | 0 | 621 |
| LG7 | 48 | 68 | 44 | 49 | 0 | 0 | 19 | 139 | 124 | 6 | 497 |
| LG8 | 58 | 120 | 45 | 86 | 11 | 12 | 0 | 156 | 225 | 32 | 745 |
| LG9 | 116 | 532 | 899 | 323 | 24 | 448 | 14 | 50 | 849 | 21 | 3276 |
| H1 | 4 | 39 | 54 | 10 | 787 | 11 | 0 | 1 | 622 | 9 | 1537 |
| H2 | 2 | 86 | 92 | 0 | 823 | 18 | 0 | 0 | 371 | 0 | 1392 |

Class abbreviations: Algae – Algae; Antil – Antillogorgia; Gvent- *G. venatlina*; SRod – Sea Rods; Apalm – *A. palmata*; Past – *P. astreoides*; Ssid – *S. siderea*; Orb – Orbicella; Rub – Rubble; Sand – Sand
