# Supplementary figures and images for: Automated classification of three-dimensional reconstructions of coral reefs using convolutional neural networks
Source: PLoS One. 2020 Mar 24;15(3):e0230671. doi: 10.1371/journal.pone.0230671 (PMC7093084; doi:10.1371/journal.pone.0230671)

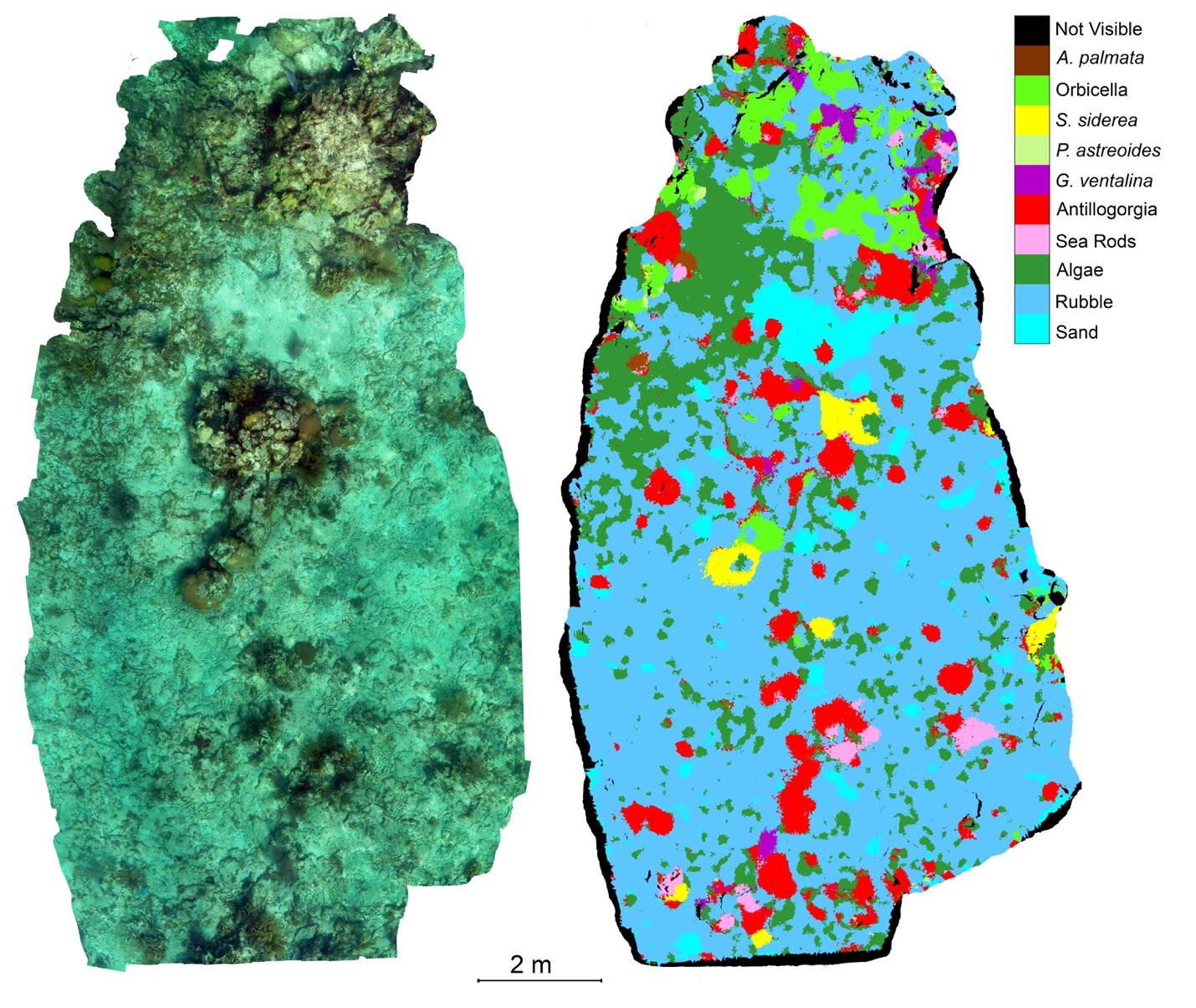

Supplement: S1 Fig — Texture mapped (left) and classified (right) overhead views of site LG1. The reconstruction was classified using nViewNet-8. (JPEG) [file pone.0230671.s002.jpeg]

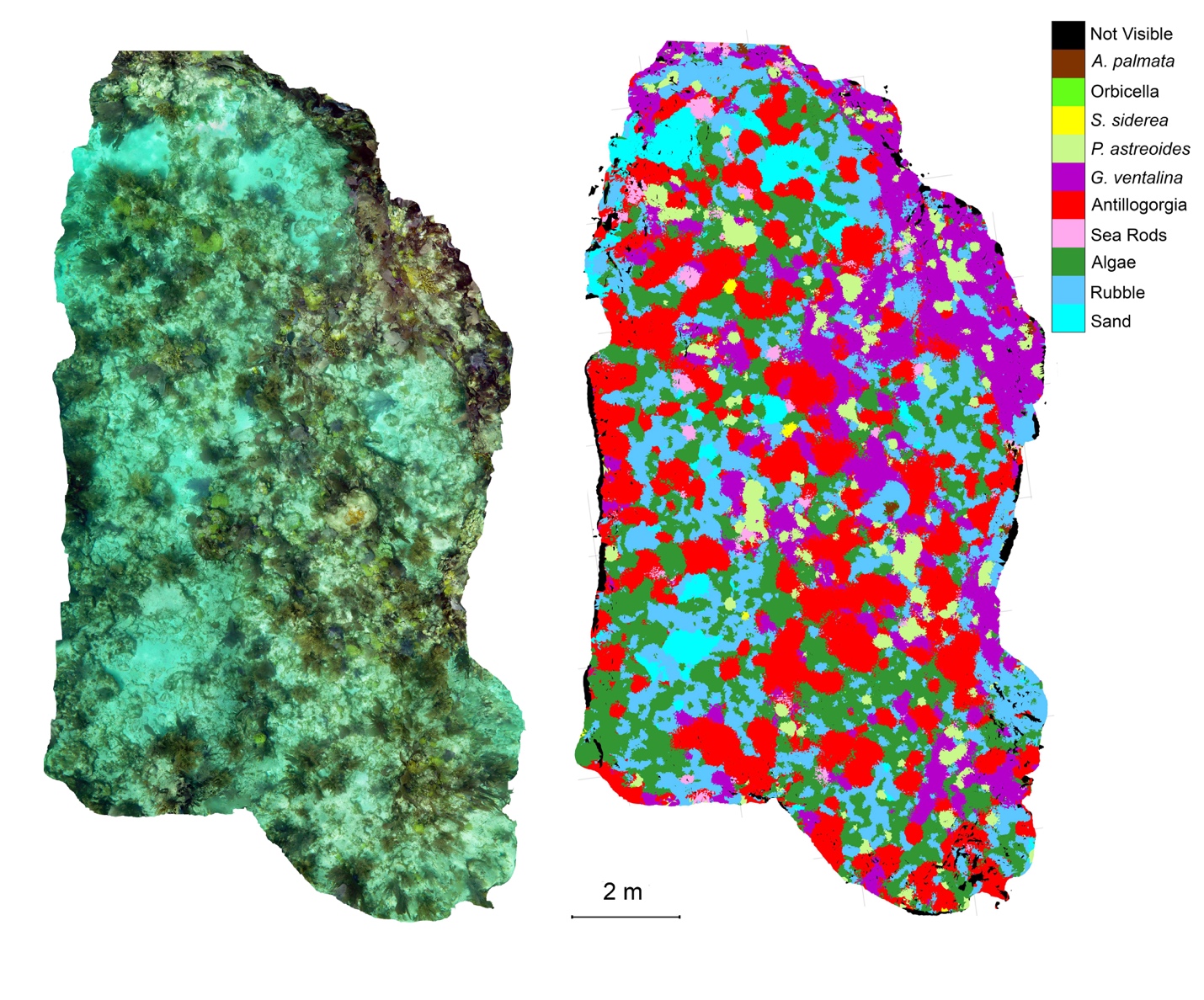

Supplement: S2 Fig — Texture mapped (left) and classified (right) overhead views of site LG3. (JPEG) [file pone.0230671.s003.jpeg]

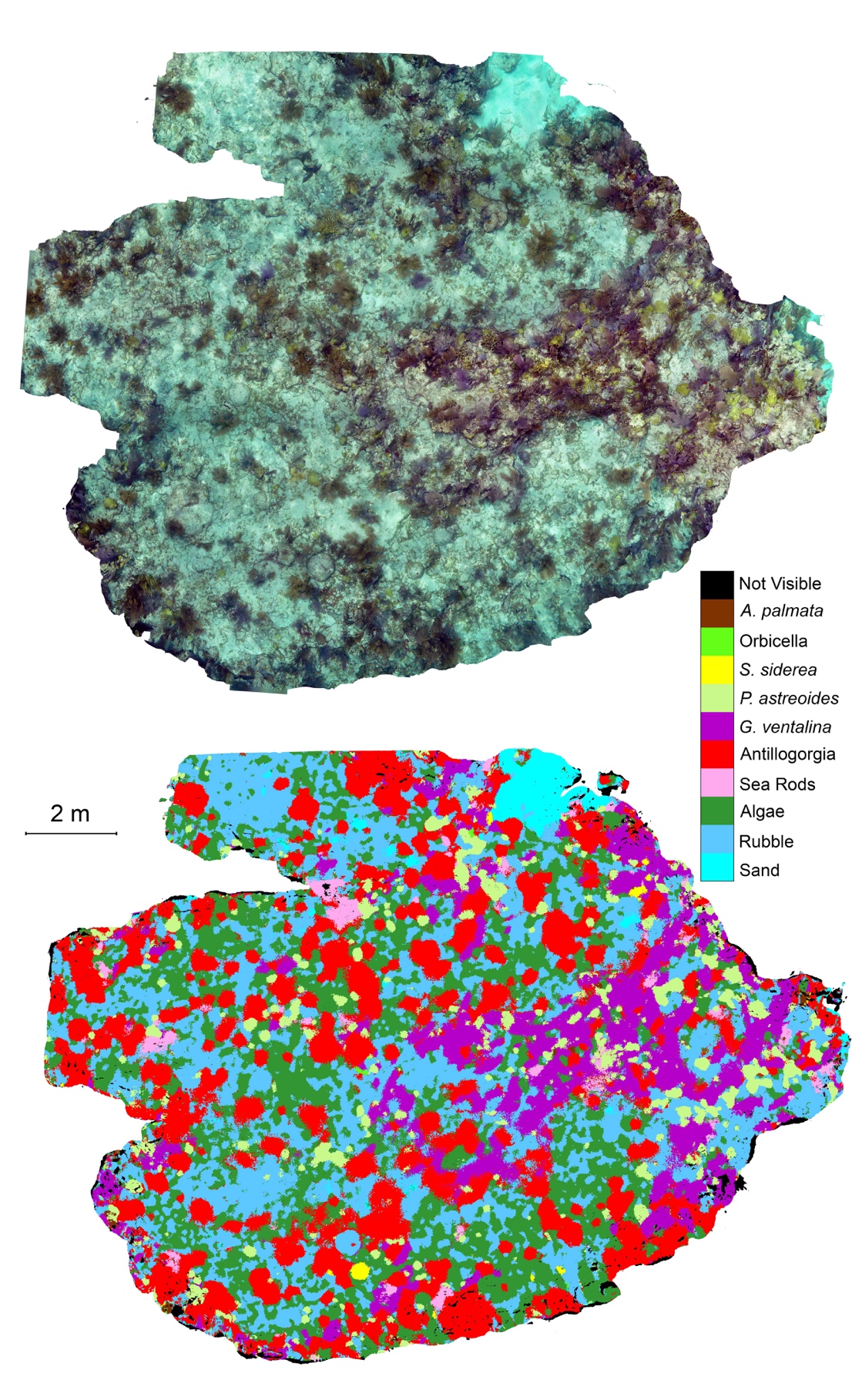

Supplement: S3 Fig — Texture mapped (upper) and classified (lower) overhead views of site LG4. The reconstruction was classified using nViewNet-8. (JPEG) [file pone.0230671.s004.jpeg]

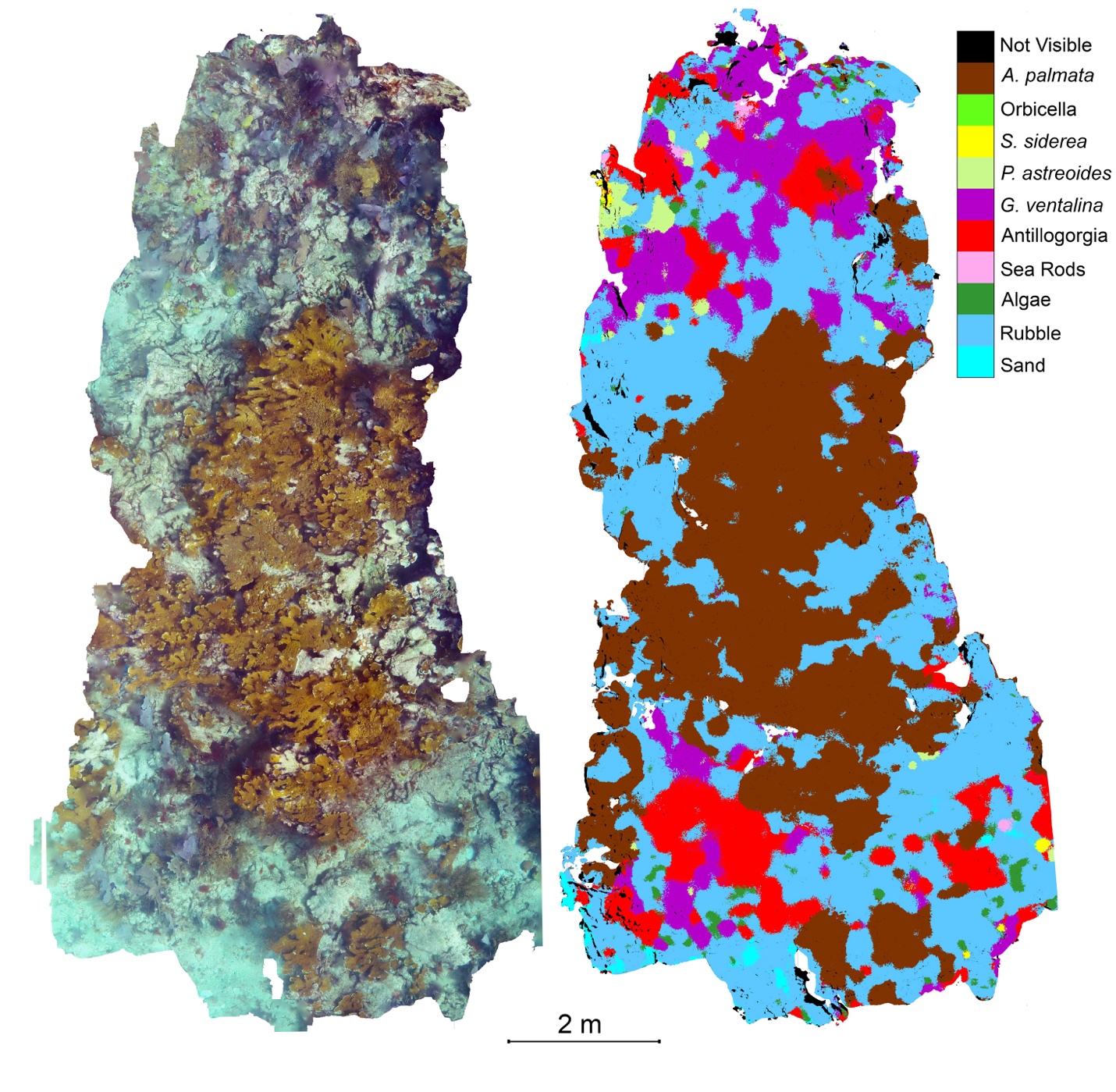

Supplement: S4 Fig — Texture mapped (left) and classified (right) overhead views of site H2. The reconstruction was classified using nViewNet-8. (JPEG) [file pone.0230671.s005.jpeg]

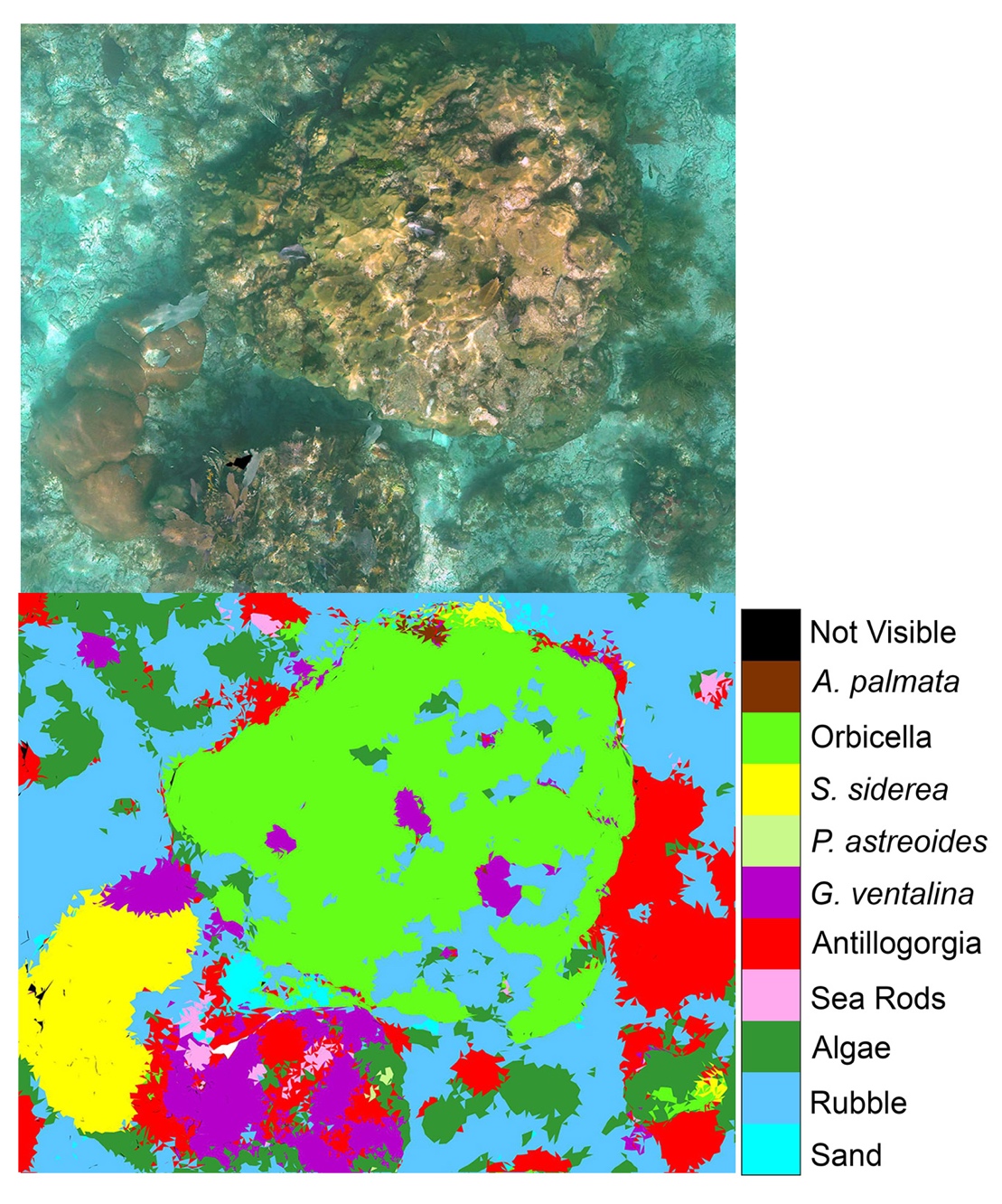

Supplement: S5 Fig — The automatic classification procedure accurately delineates the Orbicella and S. siderea colony, including distinguished live tissue from dead skeleton (labeled ‘Rubble’). The clusters of mixed octocorals and algae in the lower portion of the section (e.g. just to the right of the S. siderea colony) are more difficult to classify and result in fragmented predictions in some cases. (JPEG) [file pone.0230671.s006.jpeg]

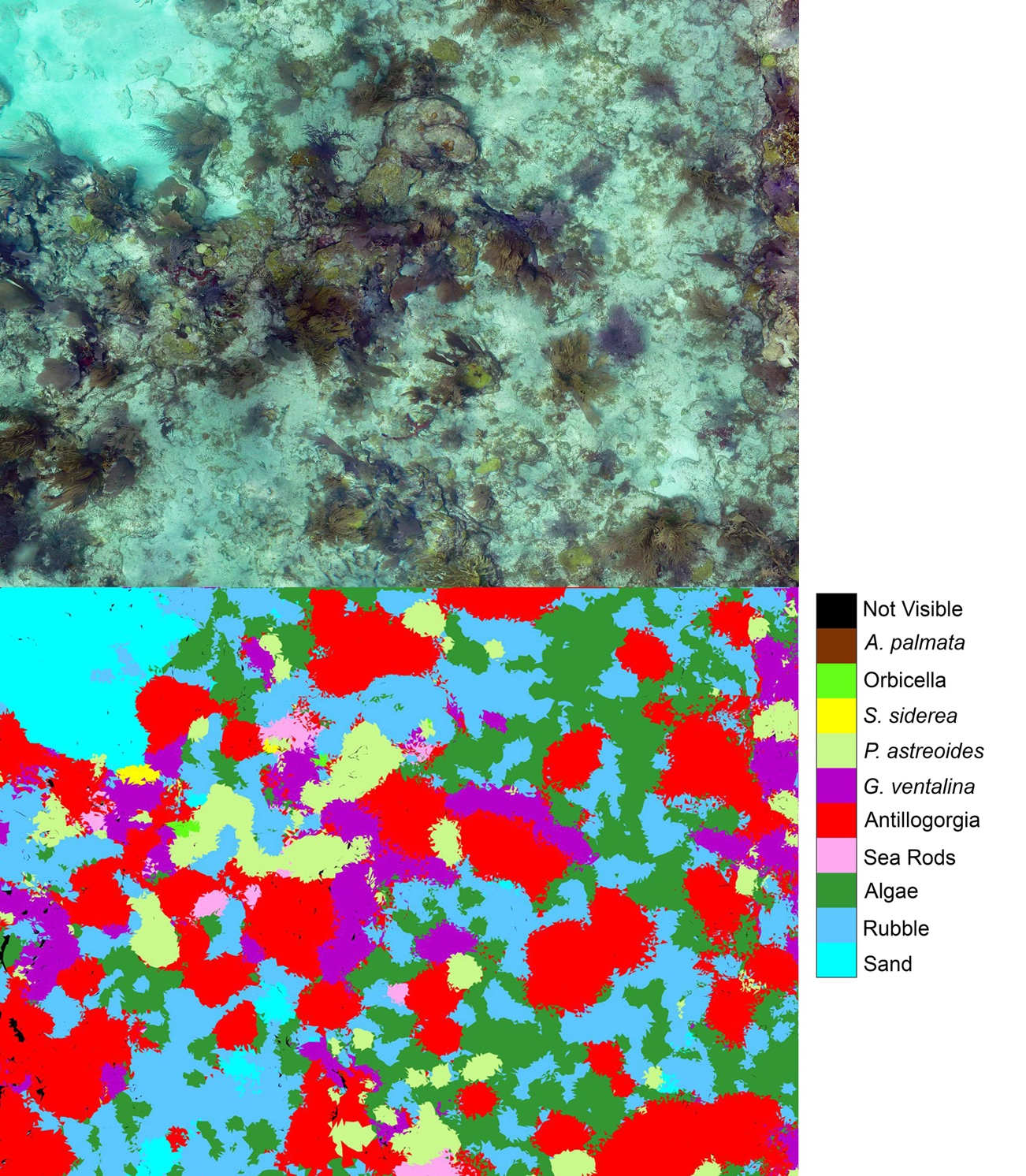

Supplement: S6 Fig — (JPEG) [file pone.0230671.s007.jpeg]
